# Contextual Barriers to Health Information Systems Optimization in Underserved Communities in Kenya: Qualitative Study Informed by Frugal Innovation and Information and Communication Technologies for Development

**DOI:** 10.2196/78950

**Published:** 2026-03-09

**Authors:** Danny Nyatuka, Md Shafiqur Rahman Jabin, Lisa Dionne-Morris

**Affiliations:** 1 Department of Medicine and Optometry, Linnaeus University, Universitetsplatsen 1, Bradford, BD7 3EN, Kalmar, 392 31, Sweden, 44 7915673612; 2 Faculty of Health Studies, University of Bradford, Bradford, United Kingdom; 3 School of Mechanical Engineering, University of Leeds, Leeds, United Kingdom

**Keywords:** contextual barriers, frugal innovation, health care delivery, health information systems, information and communication technologies for development, underserved contexts, Kenya

## Abstract

**Background:**

Health information systems (HISs) are essential for strengthening health systems in underserved areas. However, many HISs in Africa are still in the early stages of implementation, and existing systems often suffer from imbalances in data availability. Their optimization is faced with various challenges, including limited resources, which restricts their scalability.

**Objective:**

The aim of this study is to identify contextual barriers that hinder the optimization of HIS in African underserved settings. Specifically, the study adopts the lens of frugal innovation (FI) and information and communication technologies for development (ICT4D) to explore ways to enhance the quality of health care delivery for low-income populations.

**Methods:**

A qualitative research approach involving 32 participants was used. The study was guided by the central theme: contextual barriers and challenges hindering the optimization of HISs.

**Results:**

Four major thematic categories emerged from the data: HIS contextualization, health system factors, service provider issues, and HIS integration. The findings offer valuable insights that can contribute to transforming HISs in underserved settings and improving health care quality.

**Conclusions:**

The findings reflect stakeholder experiences in underserved communities in Nairobi, Kenya, and may be transferable to similar settings, subject to local governance, resources, and workflows. Despite the transformative potential of HISs in low- and middle-income countries, progress remains limited due to poor digital infrastructure and contextual barriers resulting in minimal impact from capital-intensive digital health investments and persistent data challenges. Using FI and ICT4D lenses, 4 key barriers were identified: health system, HIS contextualization, HIS integration, and HIS service provider. Rethinking HIS strategies through FI and ICT4D can enable affordable and sustainable, user-centered solutions. Future research should test scalability, sustainability, and interoperability impact in diverse settings.

## Introduction

### Background

Health information systems (HISs) are essential for strengthening health care systems in Africa by enabling timely data collection, processing, analysis, and reporting, supporting epidemiological surveillance and informing evidence-based health policy development, particularly in regions with limited infrastructure and resources [1,2]. Despite their importance, HIS implementation across African countries remains uneven, characterized by fragmented digital health initiatives and wide variations in data availability and system maturity [3,4]. While some of the countries have relatively advanced systems, many countries struggle with basic data collection, processing, and management functions, resulting in significant disparities. The adoption of key HIS interventions, including District Health Information Software 2, electronic health records, OpenMRS, mobile health, and telemedicine platforms, remains at an early stage in most African countries. Although uptake is increasing, driven largely by high mobile penetration, implementation continues to face substantial contextual barriers.

Commonly reported challenges include insufficient funding, weak health infrastructure, digital divides, limited interoperability, shortage of skilled personnel, poor data quality, restrictive policy environments, sociocultural insensitivity, limited scalability, and lack of sustained commitment beyond pilot funding phases [3-10]. Funding constraints remain a major obstacle to scaling digital health initiatives, while limited capacity to effectively use available digital tools leads to the underutilization of data and missed opportunities for improving sustainable health outcomes [6,9].

Conceptual lenses, such as frugal innovation (FI) and information and communication technologies for development (ICT4D), may offer a useful way to understand how HISs can be designed, implemented, and sustained under constraints, yet these perspectives remain under-applied in HIS research in underserved African contexts. ICT4D in health aims to bridge the digital divide in low- and middle-income countries (LMICs) by facilitating health information sharing, telemedicine, disease surveillance, health record management, computer-aided diagnosis, treatment monitoring, and health education for underserved populations [11-13]. This promotes socioeconomic development by improving health and well-being [14-16] through enabling cost-effective care, improved service quality, efficient management, enhanced access to health data, and evidence-based policy-making [17]. These benefits are realized through ICT4D principles that promote efficient, effective, and person-centered care, including user-centered design, ecosystem awareness, scalability, sustainability, data-driven solutions, open standards, privacy and security, reuse, and collaboration [18-20].

FI, on the other hand, has emerged as a novel approach to producing effective and affordable products, services, and processes that use fewer resources to improve service delivery for underserved communities [21]. FI embodies the principle of “doing more with less” [22] and is guided by simplicity, accessibility, affordability, and sustainability to create cost-effective interventions [23-25]. In health care, FI focuses on developing affordable and effective solutions in resource-constrained settings [26], responding to growing demand for cost-effective, high-quality care [27]. The limited coverage of essential health services and persistent resource scarcity, especially in rural and urban poor settings, continues to restrict access to health care [22,24,28,29]. This necessitates the need for cost-effective health care innovations and increased research in frugal health care innovation [21]. By delivering low-cost, safe, and efficient solutions, FI holds significant potential to strengthen Africa’s health systems and overcome resource constraints.

This qualitative study examines contextual barriers and challenges affecting HIS optimization in underserved communities in Kenya and identifies strategies that stakeholders perceive as feasible and locally appropriate.

### Related Works

Multiple relevant studies have been published; for example, a study to examine challenges related to the paucity of health data in Africa and its consequent impact on the implementation of digital health and evidence-based practice [6] found out that the availability of health data is limited and generally of poor quality in the African context. The challenges identified were inadequate infrastructure, a shortage of resources, and cultural barriers, which hindered efforts to leverage the potential benefits of information and communication technology (ICT) to build a properly functioning HIS. Notably, the authors observed that a lack of capacity and expertise in data analysis and interpretation led to the underutilization of available data. The study recommended automating the manual health data collection process for quick decision-making, engaging public-private partnerships to improve data collection, and investing in infrastructure and technology to enhance the collection and utilization of health data.

On the other hand, Shuvo et al [21] proposed a conceptual framework to lay out the underlying mechanisms of frugal health innovation to increase health care access by creating affordable solutions in resource-scarce settings. The study identified 3 components, that is, actors (health workers, hospitals, small and medium enterprises, research institutions, etc), the process (design, functionality, resource optimization, cost minimization, and cooperation), and the outcomes of frugal health innovation (profits from underserved customers, improved health care systems, and reverse innovation). The framework attempts to provide a blueprint for empirical research to guide managerial practices and initiate conversations on the development, adequacy, and adoption of frugal health innovations to increase affordability and access to health care for underserved populations. However, the framework is too generic, that is, focusing on frugal health innovation as opposed to digital health, and thus not fully contextualized to provide a deeper insight into the phenomenon under study.

According to the literature, the key contextual barriers to optimizing HISs in Africa include a lack of funding, digital infrastructure, HIS interoperability, skilled workforce, standard policy or guidelines, and sociocultural differences. To address these issues, previous studies have recommended increased funding, training health personnel on digital health, and engaging stakeholders in the design and development of HIS platforms, which requires more resources that are still unavailable, creating the gap this study seeks to address [30]. In addition, according to the literature, the lens of FI and ICT4D are underutilized in digital innovation in Africa, the health sector included [31-34], and particularly in Kenya’s digital health infrastructure, HIS platforms included. Furthermore, suggested digital health frameworks are more generic rather than context-specific, thus limiting insight into the research phenomena.

Therefore, a multifaceted approach to building resilient HIS is critical to strengthening health systems in LMIC settings, such as Africa. This means integrating and leveraging the potential of FI and ICT4D strategies to improve health care delivery for underserved African populations. This will help to address predominant environmental, social, and economic issues hindering health care delivery in LMICs, which contribute to the poor quality of health services [35,36].

### Study Aim and Research Questions

The study aims to identify key contextual barriers and challenges hindering the optimization of HISs by underserved communities in LMICs, with a focus on Kenya. This will inform appropriate strategies for designing and developing effective HIS platforms to overcome these challenges, thus contributing to transforming health systems to improve population health outcomes. The research questions to be answered by the study include the following:

1. What contextual barriers and challenges hinder HIS optimization in underserved communities in Kenya?
2. What strategies could be leveraged to deliver effective HIS platforms and strengthen health systems in this setting?

## Methods

### Study Setting

The study setting is urban-poor settlements, that is, Nairobi City County slums in Kenya, where residents lack basic services, which was conducted between March and July 2023.

According to Elton B Stephens Company [37], the city’s total population was estimated at 5.3 million residents as of January 2025. The majority of the people live in the slums, which lack adequate social amenities, including health services, and are hence considered underserved communities. The city hosts Kibera slums, one of the largest slums in Africa, comprising 12 villages, with an estimated population of between 235,000 and 270,000 people [38]; therefore, slum dwellers were selected as the target population for the study. The study sought to identify contextual barriers hindering the optimization of HIS interventions in underserved contexts within an LMIC setting.

### Ethical Considerations

Ethical approval for this study was obtained from the University of Leeds Research Ethics Committee. Ethical clearance was obtained from the Engineering and Physical Sciences joint Faculty Research Ethics Committee (Ref: MEEC 21‐029). Ethical issues concerning research participants, the researcher, and the sponsoring organization were addressed. These requirements were observed diligently throughout the research process to avoid breaching the ethical guidelines [39,40]. Written informed consent was obtained prior to data collection. To protect privacy and confidentiality, interviews were conducted in settings that minimized third-party presence, and transcripts were deidentified (eg, removal of names and other direct identifiers) prior to analysis. Data were stored on password-protected devices/servers with access restricted to the research team. Participants received no compensation; however, they received a reimbursement of Kshs. 1000 (US $7.7) for their time and/or travel expenses.

### Study Design

#### Overview of the Study

We conducted a qualitative study using semistructured interviews to explore contextual barriers to optimizing HIS in underserved communities in Nairobi City County, Kenya. This approach is appropriate for generating the in-depth understanding of stakeholders’ experiences, perceptions, and recommendations in real-world implementation settings [41].

The theoretical theme of “contextual barriers” was adopted to guide the study design. Benefits of the qualitative research approach include its flexibility and adaptation of interview questions for clarity as the research progresses, appropriateness for a small sample or samples, and ability to collect and interpret nonverbal cues (body language) and offer opportunity clarifications for the researcher to gain a deeper understanding of the research phenomenon [42]. The approach is therefore considered appropriate for the purposes of probing participants’ perceptions, attitudes, opinions, myths, and beliefs [43].

#### Sampling Procedure

Participants were recruited from 4 public health facilities within Nairobi slums, including Mathare North, Embakasi, Eastleigh, and Kibera South Health Centers.

##### Eligibility and Sampling

Various stakeholders were engaged. Community members and community health workers (CHWs) were eligible if they were aged 18 years or above, resided or served in the catchment areas of the participating facilities, and were willing to participate. Professional participants, including facility heads, health professionals (HPs), ICT experts, and policymakers, were eligible if they held roles relevant to HIS implementation or use, had at least 3 years of experience in their respective positions, possessed a minimum educational qualification of a diploma, and were working at the selected study sites during the study period [44]. Purposive sampling was used to ensure representation across stakeholder groups and facility types, complemented by snowball sampling for recruiting community members through CHWs.

##### Recruitment Procedures

Potential participants were contacted either in person or by phone with the assistance of the facility administrators. Interested individuals received an information sheet outlining the study aims, procedures, potential risks and benefits, privacy protections, and the voluntary nature of participation. Interviews were scheduled at participants’ convenience and conducted at the study sites.

Snowball sampling technique was used to recruit initial community members with the assistance of CHWs, who were well acquainted with their communities [45]. Purposive sampling was guided by insights from facility administrators to ensure the selection of HP respondents with appropriate characteristics for addressing specific research questions and validating findings [46]. A total of 32 participants were recruited, comprising 15 professionals and 17 community members. The professional participants’ category comprised 4 CHWs, four facility heads, 4 HPs, 2 ICT experts, 4 CHWs, and 1 policymaker.

### Data Collection and Analysis

Interview transcripts were analyzed using thematic analysis following established procedures for systematically identifying patterns and themes in qualitative data [47]. Coding was iterative and involved developing initial codes, grouping codes into categories, and refining themes through the repeated comparison of data segments and analytic memos [48]. We sought to enhance analytic rigor through the clear documentation of coding decisions and theme development [49].

The data were collected using a semistructured interview guide, which lasted between 30 and 40 minutes. The thematic analysis method was performed manually, using the streamlined codes-to-theory model for qualitative inquiry [50], and participants’ identities were coded for the purposes of anonymity. Data saturation was achieved with 25 after responses began to repeat; therefore, the data collection process was stopped. Table 1 is a sample summarization showing how initial codes were generated from ICT experts’ responses:

**Table 1.** Example of initial coding and theme development from interview transcripts (ICT^a^ stakeholder interviews; underserved communities in Nairobi City County, Kenya).

Participant ID	Main points from participant responses	Initial codes^b^	Theoretical theme^c^
ICT001	‘‘There is lack of technical skills … and high technology and maintenance costs.”	Lack of technical skills/high maintenance costs	Information management challenges
ICT001	“Donor effect creates a silo for example some partners only cater for HIV reporting and thus creating parallel reporting systems alongside the national health reporting system (DHIS^d^).”	Donor effect and parallel systems	Information management challenges
ICT001	“There is a problem with policy implementation especially on devolution of health and misappropriation of funds.”	Poor policy implementation	Information management challenges
ICT004	“ICT infrastructure is expensive … that is, there is high technology and maintenance costs.”	Expensive ICT infrastructure	Information management challenges
ICT004	“The HR^e^ element should be looked at, that is …, there is low developer capacity and technical skills that is, developers do not understand execution of systems.”	Low HIS^f^ developer capacity	Information management challenges
ICT004	“There is lack of clear objectives program characterised by short-term thinking … and … a lot of bureaucracy that policy people are not open with new technologies but they only favour paperwork.”	No long-term strategy on sustainability of HIS projects	Information management challenges
ICT004	“There is a lot of bureaucracy that policy people are not open with new technologies but they favour paperwork.”	A lot of bureaucracy	Information management challenges

a ICT: information and communication technology. Refers to participants with ICT expertise.

b Initial codes: shortened or synthesized statements capturing the key idea expressed in participant responses. Multiple ideas from a single response may be separated by a slash (/) to indicate combined coding.

c Theoretical theme: higher-level category used to group similar initial codes reflecting common patterns or challenges in HIS implementation.

d DHIS: District Health Information System, the national health reporting platform in Kenya.

e HR: health record.

f HIS: health information system.

Table 2 summarizes how the categories and data themes were generated from the initial codes derived from ICT experts’ responses. Four key data themes emerged, namely service provider, HIS integration, health system, and HIS contextualization.

**Table 2.** Generating categories and data themes from initial codes extracted from information and communication technology (ICT) experts’ responses.

Theoretical themes	Initial codes^a^	Categories or subcategories^b^	Data themes^c^
Information management challenges	Lack of technical skills and high maintenance costs	Lack of expertise; high costs	Service provider
Information management challenges	Donor effect and parallel systems	Parallel systems (silos)	HIS^d^ integration
Information management challenges	Poor with policy implementation	Management and leadership	Health system
Information management challenges	Expensive ICT infrastructure	Unaffordable infrastructure	HIS contextualization
Information management challenges	Low HIS developer capacity	Technical expertise	Service provider
Information management challenges	No long-term strategy on the sustainability of HIS projects	Management and leadership	Health system
Information management challenges	A lot of bureaucracy	Management and leadership	Health system

a Initial codes: key points extracted from participant responses.

b Categories or subcategories: groups of related codes reflecting common concepts.

c Data themes: overarching themes derived from categories, representing major patterns in the findings.

d HIS: health information system.

We aligned the study design with the COREQ (Consolidated Criteria for Reporting Qualitative Research) checklist, appropriate for interview-based qualitative studies [51,52]. The completed checklist is as a supplementary file (Checklist 1) upon resubmission, in accordance with JMIR recommendations and EQUATOR guidelines.

## Results 

This section presents the study findings on contextual barriers and challenges affecting HIS optimization in underserved communities. Four themes were identified: (1) health system factors, (2) HIS contextualization, (3) HIS integration, and (4) service provider factors.

### Health System

Participant responses around this theme comprised issues related to health care policy decisions, governance, financing, resource provision, and implementation of national projects, including HIS. Sarkar defines a health system as a network of people, institutions, facilities, communities, and resources provision, including health personnel, who perform various tasks and provide health services to citizens, hence collective responsibility [53]. Key stakeholders included government (policymaking and financing), citizens (health care consumers), health care providers (hospitals and HPs), and development partners, among other stakeholders. However, it is the sole responsibility of the government to provide policy guidelines, ensuring a standard approach toward the delivery of health services, including HIS projects. The study identified various concerns during the study, most of which pointed to various weaknesses that act as barriers to successful health systems, including HIS operations. The following are quotes extracted from some of the participants’ responses under this theme:

*Regarding management of information and records, we mainly emphasise on professional training and code of conduct otherwise no organisational policy is in place to guide management health information including during referral.*

[AP005]

*There is lack of clear objectives characterised by short-term thinking … and a lot of bureaucracy that policy people are not open with new technologies but they only favour paperwork.*

[ICT004]

*Information management policies used in this place are based on WHO guidelines.*

[AP002]

*There is a problem with policy implementation especially on devolution of health and misappropriation of funds.*

[ICT001]

*There is poor retrieval of files, scarcity of storage space, and confidentiality of patient data especially Mother/Child booklet, misplacing of patient files … etc.*

[HP003]

The study noted weaknesses in the health system, including a lack of standard policy guidelines regarding managing health information practices and services provided in public health facilities. Some of the facilities had no clear procedure for creation, storage, and retrieval and sharing of patient records or information during a referral. It was reported that World Health Organization information management policies were being followed, which did not consider contextual factors to meet local information users’ needs. Health records were mainly managed manually due to a lack of digital infrastructure, which made retrieving records difficult; some records were disposed of prematurely, while some went missing, leading to information loss. In addition, participants reported poor policy implementation procedures in devolution regarding specific functions of both the national government and the county government, which led to poor health care funding in the country. Most of these challenges are because of resource scarcity.

### HIS Contextualization

Some of the participants reported on technology-related issues that needed to be contextualized to meet local needs through an understanding of the technological environment. The digital era is becoming increasingly dynamic, while the needs of HIS users are equally increasing and changing quickly. According to observations made during the study, health care organizations need to adopt context-specific HIS platforms to ensure their meaningful use and increase operational efficiency. Unfortunately, most HIS in Africa lack contextualization, but they are rather based on foreign designs, and thus, they fail to respond to the needs of user systems, thereby compromising the quality of health care delivery. The following are quotes from the respondents that relate to this theme:

*ICT infrastructure is expensive … there is high technology and maintenance costs.*

[ICT004].

*The existing system for nutrition does not meet needs of the nutritionist. The system cannot generate reports so I need a tool to analyse and generate reports.*

[HP006].

*You should ascertain stakeholder requirements and map user needs first.*

[ICT001]

*Customise software to suit users’ needs for example Open MR software for doctors and nurses.*

[ICT002]

*Localise and customise the system to allow development of own skills.*

[ICT001]

*Use computers that is, laptops and desktops, servers, IoT to capture data and mobile phone for patients however … mobile phones are not meant for doctors but only patients.*

[ICT001]

*One should consider configurations for offline systems to enable data collection when Internet fails and ensure unique user identification.*

[ICT003]

*Consider affordability and quality of the technological infrastructure itself.*

[ICT004]

The above extracts from participants’ responses confirm the findings regarding the need for HIS contextualization in terms of the nature of the technical infrastructure, that is, considering technology acquisition and maintenance costs to make it affordable and accessible and customizing, configuring, and localizing software and hardware systems to meet different users’ or stakeholders’ needs to ensure the meaningful use to promote operational efficiency [54]. However, the existing digital infrastructure is unaffordable in underserved contexts due to resource limitations and high costs of technology; therefore, there is a need to combine and leverage ICT4D and FI principles to contextualize HIS. Unfortunately, the people’s voices have not been heard concerning their health and well-being, but they are instead treated as passive receivers rather than active participants [55].

### HIS Integration

The participants raised concerns about HIS integration across the board, which, in their opinion, was critical in enhancing national health care collaboration. A major barrier reported to have seriously hindered the optimization of HIS in an underserved context was the existence of data silos at the institutional level [56]. The silos were mainly observed to be in the interest of the donor rather than those of the government and the people (citizens). This raised serious concerns about data access by users, including personal health data by patients and HPs. This greatly hindered the care decisions, especially in situations where the patient received care in more than 1 facility, a situation that would even lead to medical mistakes due to the unavailability of pertinent information. The following are extracts from the participants to confirm this observation:

*We have manual health information services as there is no government IT infrastructure except that of partners … for example university of Maryland transmit some data online*. *This creates loopholes in the health care system hence parallel health reporting which compromises efforts toward health care collaboration to improve service delivery*.

[AP003].

*HIS integration will require specific eHealth standards such as HL7 and FHIR especially in facilities with systems already installed.*

[ICT002].

*The donor effect creates a silo, for example, some partners only cater for HIV reporting and thus creating parallel reporting systems alongside the national health reporting system (DHIS).*

[ICT001].

*Use IT to integrate information systems, and recruit adequate personnel.*

[HP009]

*The impact of ICT is still low since only mobile phone is used to communicate especially during emergency.*

[AP002]

The study observed that government partners and sponsors (nongovernmental organizations) established their information infrastructure in public health facilities to support reporting on their project activities or programs, without necessarily aligning it with the government’s strategic plan. In most of the facilities, partners owned the entire digital information infrastructure, including computing equipment, software, ICT personnel, commodities, and so forth, to support their programs without necessarily involving government employees. Numerous challenges related to data access were observed during the study, particularly on health data security, client privacy, and confidentiality due to the nature of the information practices, thereby violating the individual’s right to privacy and confidentiality. This is because information was mainly managed manually, resulting in the lack of proper mechanisms to safeguard privacy and confidentiality. Communication and sharing of information between patients and HPs were mainly done via cell phones, which were not inclusive and had numerous limitations, including small data processing and storage capacity as well as small screens, which could not be effective in an office setup.

### HIS Service Providers

Service providers refer to individuals or organizations that receive support from the government to offer various health services to clients or citizens. The service provider is responsible for planning and controlling operations, capacity building, service improvement, and resource allocation in individual health care organizations. The participants’ responses revolved around dissatisfaction with service provision to clients, ICT services included. The lack of ICT infrastructure, basic medical equipment, electricity, stationery, and sitting space was a serious concern, leading to clients’ dissatisfaction with services rendered.

The following are quotes extracted from participants’ responses regarding ICT infrastructure that provides HISs:

*Key factors contributing to the nature of current information services are understaffing, lack of ICT infrastructure, lack of staff training, resistance to use of computers, lack of knowledge or benefit of IT*.

[AP005]

*Photocopy machine and computers are available; however, computers are not used at all. Only the facility mobile phone is used by staff.*

[HP011]

*Staff are reluctant! And they feel its extra work to use computers since we do not have adequate personnel, lack of training and computer literacy! Health workers have a phobia with computers … and lack of equipment maintenance.*

[AP001]

*Information services can be improved by automation and training staff in IT literacy.*

[AP002]

*Improve ICT infrastructure, create awareness/training of staff in ICT and ensure global automation of departments’ information services.*

[AP005]

*More personnel should be hired, funding for training of ICT skills be provided, and creation of more space for training and keeping records.*

[AP003]

*Implement ICT fully to bring transparency towards improving health care delivery.*

[AP001]

Participants’ accounts, therefore, suggest that the limited managerial prioritization of ICT implementation, combined with resource constraints and training gaps, contributed to low uptake of available digital tools. Most facilities had no HIS infrastructure due to a lack of the necessary equipment and skilled workforce. The situation was attributed to a lack of understanding of the value of ICT in their work activities, and thus, resistance against the quest to use computers despite having been provided by partners donating computers to some of the facilities. In addition, implementation efforts remained ineffective because essential foundational tools and services were still lacking, including reliable internet connectivity, functional electronic medical record systems, stable power supply, and ongoing technical support, thereby further limiting the practical use and sustainability of donated digital infrastructure. As a result, most of the staff were not confident in using computers due to inadequate exposure, which had become a culture, thus creating an avenue for resistance against adopting ICT. Most public health institutions in LMICs struggle to provide satisfactory services to clients due to resource limitations; therefore, adopting appropriate approaches and strategies to improve service delivery is essential.

## Discussion

### Principal Findings

This study aimed to identify contextual barriers and challenges affecting health information system optimization in underserved communities in Kenya and to explore feasible, locally appropriate strategies suggested by stakeholders. We found four interrelated themes, health system factors, HIS contextualization, HIS integration, and service provider factors, highlighting how governance, resourcing, workflow realities, and capacity constraints shape HIS use and perceived value. HISs are a critical tool for strengthening health systems in underserved contexts. Africa’s HISs are in the early stages of implementation, while those in existence have data imbalances in data availability.

The lack of power supply, digital infrastructure, internet connectivity, interoperability standards, and skilled ICT personnel hinders the sharing, communication, and/or exchange of health data for better decision-making. The lack of integration between different HIS stakeholders leads to data silos within a country, making it difficult to share health data among health care providers and facilities [4]. According to Nyatuka and De La Harpe, most African countries do not have a national health information management strategy, resulting in the uneven adoption of HIS tools by different stakeholders, which jeopardizes the compatibility of health systems and thus leads to parallel HIS reporting on unrelated indicators [57]. Most HISs in Africa are vertical, partner-driven, and program-specific due to donor influence with limited system-wide impacts, and as a result, the majority of the projects are mainly designed to track and manage specific diseases such as HIV/AIDS, malaria, and tuberculosis, rather than providing a holistic or comprehensive historical record of a patient [4,43,53,58].

Furthermore, the absence of proper policy and regulatory guidelines and standards hinders the adoption of HISs [4,5]. Without guaranteed long-term sustainability, HIS projects will always fail, becoming merely exercises in futility with unmet expected impact, regardless of the level of investment or technological sophistication involved [3]. The situation, therefore, calls for the adoption of innovative strategies that can enhance and build the capacity for HISs in underserved contexts from LMICs to meet health data requirements. The study aims to leverage the principles of FI and ICT4D (digital development) to address contextual barriers hindering the optimization of HISs in underserved contexts. This is to be achieved through an empirical investigation to identify these barriers and recommend possible ways to overcome them using the lens of FI and ICT4D.

Despite the efforts addressing the barriers to overcome the use of FI and ICT4D, many health care systems in Africa continue to experience resource constraints and the digital divide, resulting in lower ICT penetration levels, design-reality gaps, inadequate health infrastructure, and policy issues [2,59]. Furthermore, there is a lack of attention to people’s context of using technology when designing eGovernment programs, including eHealth strategies [14,60]. Noticeably, while access to ICT plays a critical role in promoting economic development, the nature and level of ICT usage do not reflect the same in most LMICs. To effectively leverage ICT4D in promoting health care, the focus should be on incorporating an FI strategy to create accessible, low-cost, and sustainable HISs, tailored to address specific local needs and contexts to reap the full benefits of ICT4D [43,61].

For example, in a study that explored the digital transformation within the health care sector in Africa, Iyawa et al [9] noted various pitfalls and challenges faced. These include a lack of digital health expertise and skills to develop and maintain digital interventions, a limited number of donors despite increasing demand on grant-seeking organizations, and disparate donor systems and standards, limiting interoperability and the exchange of various platforms. The authors also noted that the funding of digital health was lagging due to resource constraints, whereby early implementations lacked sustainability, given their cost-intensive maintenance nature. The study recommends digital health training in the training curriculum of health care practice for the digital transformation of global health in Africa.

### Implications for Practice—Health System

These findings indicate that health system governance, financing, and infrastructure constraints limiting effective HIS implementation should be addressed through FI- and ICT4D-oriented strategies, including the development of cost-effective, scalable, and sustainable digital health infrastructure and the adoption of interoperable and affordable HIS platforms. For implementers, this implies integrating ICT4D principles, including understanding the health care ecosystem, designing for scalability, building for sustainability, and fostering collaboration into HIS design and deployment [59], alongside FI principles emphasizing affordability and sustainability to ensure feasibility in resource-constrained environments [24]. For policymakers, this implies adopting and enforcing FI- and ICT4D-aligned policies to guide investment in sustainable digital health infrastructure, enable electronic health information management, and support evidence-based decision-making by health system leaders and governing authorities. Together, these approaches can mitigate persistent resource constraints by enabling affordable HIS development and improving the availability of timely, high-quality health data to meet system needs [62,63], particularly in underserved urban LMIC settings where limited infrastructure and financing hinder HIS optimization.

### Implications for Practice—HIS Contextualization

These findings suggest that HIS contextualization challenges, particularly noninteroperability and health data silos, should be addressed through FI- and ICT4D-oriented actions, including embedding these principles throughout the design, development, and implementation of HIS platforms [60-62] and adopting open-source software, interoperability standards, and open innovation approaches to support seamless workflows [63-65]. For implementers, this implies engaging end users in selecting context-appropriate technologies, prioritizing privacy, data security, affordability, and usability, and leveraging scalable and cost-efficient mobile–based and cloud-based solutions to strengthen HIS across facilities and systems [24,66]. For policymakers, this implies supporting FI- and ICT4D-aligned digital health strategies and standards that promote interoperability, sustainability, and affordability of HIS interventions, particularly in underserved urban LMIC settings where fragmented infrastructure constrains integrated health information management.

### Implications for Practice—HIS Integration

These findings indicate that HIS integration challenges, especially noninteroperability and health data silos that limit information exchange, should be addressed through FI- and ICT4D-oriented actions, including applying these principles in the design, development, and implementation of HIS platforms and adopting open-source software, interoperability standards, and open innovation to enable context-appropriate workflows [15,18]. For implementers, this implies involving end users in selecting preferred technologies, ensuring HIS-supported services comply with privacy, security, and affordability requirements and prioritizing scalable mobile–based and cloud-based solutions to strengthen cross-facility and system integration [50,54].

For policymakers, this implies promoting FI- and ICT4D-aligned digital health strategies, interoperability frameworks, and investment models that support sustainable, affordable, and integrated HIS infrastructure, particularly in underserved urban LMIC settings where fragmented digital architectures constrain effective health information exchange [67].

### Implications for Practice—HIS Service Provider Theme

These findings suggest that service provider-level constraints in accessing and sustaining digital health infrastructure should be addressed through the deliberate adoption of FI and ICT4D strategies, including open-source software (eg, OpenMRS), open data, open standards, and reuse of existing systems to develop cost-effective HIS platforms [20,68,69]. For implementers, this implies that health facility managers and governing authorities should improve access to digital health resources by reducing infrastructure and maintenance costs and establishing internal policies and procedures aligned with FI and ICT4D principles to support sustainable HIS implementation. For policymakers, this implies developing national and county-level digital health frameworks that incentivize affordable and sustainable HIS investments and strengthen institutional capacity for digital transformation, particularly in underserved urban settings where resource limitations hinder access to reliable health information systems.

### Strengths and Limitations of the Study

We enhanced the trustworthiness of the analysis by maintaining an audit trail of coding and theme development, using iterative comparison across transcripts and documenting analytic decisions. We provide a thick description of the study setting to support the assessment of transferability. The findings should be interpreted in light of the urban informal settlement context of Nairobi City County.

The study’s empirical findings offer evidence-based insights into complex HIS development challenges in an underserved setting, such as Africa, and Kenya in particular, leading to the delivery of context-specific solutions. Additionally, the identification of key contextual barriers and proposing the integration of FI and ICT4D strategies as interpretive lenses into the development process of HIS interventions provides valuable insights for digital health researchers and stakeholders to address resource limitation challenges more appropriately.

The study’s findings, based on a case study of Kenya, may not be generalizable to other regions due to differing demographics, sociocultural factors, and digital health infrastructures. Limited research on frugal innovation strategies in African digital health contexts also constrained the study’s literature base. Future research should use mixed methods to provide deeper, more valid insights into the complex issues studied.

### Conclusion

The findings reflect stakeholder experiences in underserved communities in Nairobi City County, Kenya. While some barriers (eg, infrastructure constraints and parallel reporting systems) may be relevant to similar settings, transferability should be assessed in relation to local governance, resources, and workflows.

The study found out that despite the transformative potential of HISs in underserved LMIC settings, progress remains limited due to poor digital infrastructure and contextual barriers. Capital-intensive digital health investments have produced minimal impact, contributing to data quality challenges and weakened evidence-based decision-making. Using FI and ICT4D lenses, 4 key barriers were identified: health system, HIS contextualization, HIS integration, and service provider.

The findings indicate that rethinking HIS implementation strategies is necessary to improve sustainability and effectiveness. FI and ICT4D offer practical approaches for developing affordable, user-centered, and resilient HIS interventions that can strengthen health systems and promote equitable health care access in underserved communities.

Future studies should test FI and ICT4D informed HIS solutions in diverse settings to assess scalability, sustainability, and effects on data interoperability and health system decision-making.

## Supplementary material

10.2196/78950 Checklist 1 COREQ checklist.

## Abbreviations

CHW: community health worker
COREQ: Consolidated Criteria for Reporting Qualitative Research
FI: frugal innovation
HIS: health information system
HP: health professional
ICT: information and communication technology
ICT4D: information and communication technologies for development
LMIC: low- and middle-income country
