# Supplementary material for: Contextual Barriers to Health Information Systems Optimization in Underserved Communities in Kenya: Qualitative Study Informed by Frugal Innovation and Information and Communication Technologies for Development
Source: JMIR Form Res. 2026 Mar 9;10:e78950. doi: 10.2196/78950 (PMC12978921; doi:10.2196/78950)
Supplement: Checklist 1 [file formative-v10-e78950-s001.pdf]

Multimedia Appendix 1: COREQ Checklist – Interview-Based Qualitative Study

	**Research Team and Reflexivity**	
**S/N**	**Guide Question**	**Response**
**1**	Who conducted the interviews?	The interviews were conducted by the lead researcher, assisted by a research assistant, who also assisted in recruitment, data collection, and analysis.
**2**	What are the interviewer's credentials and experience?	The interviewer is a Lecturer at Strathmore University**,** with experience in qualitative research and health information systems studies. Specifically, trainings attended include:<br>• Thematic Analysis or Coding Workshops – training on coding qualitative data, generating themes, and using manual or software-assisted approaches.<br>• Research Ethics in Human Subjects Research – training on obtaining informed consent, confidentiality, and ethical data handling by TRREE.
**3**	Was there a prior relationship with participants?	There was no prior personal relationship with participants. Participants were introduced to the study through facility administrators and CHWs.
**4**	What did participants know about the researcher?	Participants were informed that the researcher was **a** university lecturer conducting a study on Health Information Systems (HIS)**.** They were also provided with an information sheet outlining the study aims, procedures, voluntary participation, and privacy protections**.**
**5**	Were researcher biases or characteristics considered?	The researcher maintained reflexivity by being aware of potential biases, such as prior knowledge of HIS and digital health systems, and took measures to ensure objective questioning and consistent coding during analysis.
	**Study Design**	
**6**	What qualitative approach or theoretical	A qualitative research approach was adopted, guided by the theoretical theme: *"contextual barriers & challenges hindering optimization of Health Information*

	framework guided the study?	*Systems (HIS)"*. This framework allowed exploration of participants' perceptions, attitudes, and experiences regarding HIS implementation in underserved urban settings.
**7**	How were participants selected and recruited?	• Purposive sampling was used to select professional participants (facility heads, health professionals, ICT experts, policymakers) based on their roles in HIS implementation.<br>• Snowball sampling was employed to recruit community members through community health workers (CHWs), who were familiar with the local communities.<br>• Recruitment was facilitated by facility administrators who contacted potential participants either in person or by phone.
**8**	What were the eligibility criteria and sample size?	• Community members/CHWs**:** aged ≥18 years, resided or worked in the catchment areas of participating facilities, and willing to participate.<br>• Professional participants: held roles relevant to HIS implementation or use, had at least three years' experience, a minimum educational qualification of a diploma, and were working at the selected study sites.<br>• Sample size: 32 participants (15 professionals, 17 community members).
**9**	Where and how were interviews conducted?	• Interviews were conducted at the selected public health facilities in Nairobi slums (Mathare North, Embakasi, Eastleigh, Kibera South).<br>• Settings were chosen to minimize third-party presence and protect participant privacy.<br>• Interviews were conducted at times convenient to participants, and written informed consent was obtained prior to data collection.
**10**	Was an interview guide used?	Yes. A semi-structured in-depth interview guide guide was used.

11	Were interviews recorded, and what was their duration?	The interviews were recorded, and they lasted between 30 and 40 minutes each.
12	Was data saturation achieved?	Yes, data saturation was reached after 25 interviews**,** when responses began to repeat, after which data collection was stopped.
	**Data Analysis**	
13	How was the data coded and analyzed?	Data were analyzed using thematic analysis (TA) manually. The researcher extracted initial codes, categories, and subcategories from participants' responses, which were then used to identify broader patterns and themes. The streamlined codes-to-theory model guided the coding process.
14	How were themes derived from the data?	Themes were derived inductively from the initial codes. Codes were grouped into categories and subcategories, which were then synthesized into four main themes**:** *service provider, HIS integration, health system, and HIS contextualization*.
15	How many researchers were involved in coding?	Coding was conducted primarily by the lead researcher (single-coder analysis).
16	Was software used for analysis?	No qualitative analysis software was used; the analysis was performed manually**.**
	**Study Findings and Trustworthiness**	
17	Are participant quotations presented to support themes?	Yes, participant quotations are provided, particularly from ICT experts, to illustrate initial codes and support the identification of themes.
18	Is there consistency between data, codes, and findings?	Yes, the codes, categories, and subcategories are clearly linked to the derived themes, demonstrating consistency between raw data, coding, and study findings.
19	Were findings validated by participants (member checking)?	No, participant validation (member checking) was not conducted**.** Participant validation (member checking) was not conducted due to the sensitive nature of the discussions, the logistical challenges of re-contacting participants across multiple urban-poor facilities, and the focus on professional and community stakeholder perceptions that were systematically analyzed. Credibility and trustworthiness were

		ensured through systematic coding, use of a clear thematic framework, detailed audit of codes and categories, and linking all findings to direct participant quotations, providing transparency and consistency in the analysis.
	**Ethics**	
20	Was ethical approval obtained?	Yes, ethical approval was obtained from the University of Leeds Research Ethics Committee, specifically the Engineering and Physical Sciences Faculty Research Ethics Committee (EPS FREC) (Ref: MEEC 21-029).
21	How was informed consent obtained?	Written informed consent was obtained from all participants prior to data collection. Participants were provided with an information sheet describing the study aims, procedures, potential risks and benefits, privacy protections, and the voluntary nature of participation.
22	How was confidentiality and data security maintained?	• Confidentiality was maintained by conducting interviews in settings that minimized third-party presence and de-identifying transcripts (removing names and direct identifiers).<br>• Data were stored on password-protected devices and servers, with access restricted to the research team.
